# Diabetic Retinopathy Features Segmentation without Coding Experience with Computer Vision Models YOLOv8 and YOLOv9

**DOI:** 10.3390/vision8030048

**Published:** 2024-08-23

**Authors:** Nicola Rizzieri, Luca Dall’Asta, Maris Ozoliņš

**Affiliations:** 1 Department of Optometry and Vision Science, Faculty of Physics, Mathematics and Optometry, University of Latvia, Jelgavas Street 1, LV-1004 Riga, Latvia; 2 Research and Development, LIFE Srl, IT-70100 Bari, Italy; 3 Institute of Solid State Physics, University of Latvia, Kengaraga Street 8, LV-1063 Riga, Latvia

**Keywords:** diabetic retinopathy, computer vision, segmentation, YOLOv8, YOLOv9, retinal fundus

## Abstract

Computer vision is a powerful tool in medical image analysis, supporting the early detection and classification of eye diseases. Diabetic retinopathy (DR), a severe eye disease secondary to diabetes, accompanies several early signs of eye-threatening conditions, such as microaneurysms (MAs), hemorrhages (HEMOs), and exudates (EXs), which have been widely studied and targeted as objects to be detected by computer vision models. In this work, we tested the performances of the state-of-the-art YOLOv8 and YOLOv9 architectures on DR fundus features segmentation without coding experience or a programming background. We took one hundred DR images from the public MESSIDOR database, manually labelled and prepared them for pixel segmentation, and tested the detection abilities of different model variants. We increased the diversity of the training sample by data augmentation, including tiling, flipping, and rotating the fundus images. The proposed approaches reached an acceptable mean average precision (mAP) in detecting DR lesions such as MA, HEMO, and EX, as well as a hallmark of the posterior pole of the eye, such as the optic disc. We compared our results with related works in the literature involving different neural networks. Our results are promising, but far from being ready for implementation into clinical practice. Accurate lesion detection is mandatory to ensure early and correct diagnoses. Future works will investigate lesion detection further, especially MA segmentation, with improved extraction techniques, image pre-processing, and standardized datasets.

## 1. Introduction

Diabetes is a severe and chronic disease characterized by high blood glucose levels [1,2]. It is a leading cause of death and disability worldwide and affects people regardless of gender, age, and birthplace. According to estimates from the 2019 Global Burden of Diseases, Injuries, and Risk Factors (GBD) Study, diabetes was the world’s eighth-leading cause of death and disability, with approximately 460 million people living with the disease [3]. In 2021, the International Diabetes Federation (IDF) estimated that approximately 537 million people worldwide had diabetes [4], representing a substantial and recognized problem for healthcare systems [5]. In 2023, the updated GBD 2021 was published [6], which estimated that 529 million people of all ages had diabetes (a global prevalence of 6.1%). By 2050, the global prevalence of diabetes will increase by 60%, reaching 9.8% (approximately 1.31 billion people worldwide). The form of diabetes that will most represent the global diabetic population is Type 2, i.e., with a solid genetic characterization and a strong association with a sedentary lifestyle and obesity. Males and those over 65 years old are at a higher risk, according to the same report.

Diabetic eye disease, caused by damage to the retinal capillaries, is the most common microvascular end-organ complication of diabetes and includes diabetic retinopathy (DR) and macular edema (ME) [7]. DR occurs in 30% to 40% of all diabetic individuals, and the increasing prevalence of diabetes parallels DR [8]. A recent systematic review and meta-analysis accounting for 59 population-based studies published up to March 2020 [9] showed that 103 million people are suffering from DR, and these estimates will further increase to 161 million individuals by 2045. Based on other short-term projections for 2030, DR prevalence is increased in middle- and low-income countries, ranging from 20% to 48%, far above the rates in high-income countries. The DR pandemic is a pressing global issue that must be urgently addressed [10].

DR can be described as non-proliferative (NPDR), proliferative (PDR), and maculopathy or macular edema (ME). NPDR is known as background DR, whereas PDR and ME represent its advanced stages [11,12]. DR patients can show different signs, such as micro-aneurysms (MAs), hard exudates (HEs), hemorrhages (HEMOs), and cotton wool spots (CWSs) in varying stages of the disease. MAs are weak dark red spots developed on blood vessels that bulge outward. They are the first detectable change in the retina due to DR. Bleeding, or a HEMO, is dark red, usually round or oval, and formed by an MA rupture. HEs and CWSs are collectively known as exudates (EXs). The first are yellowish deposits of protein in the retina. The latter, which are softer, are white and fluffy lesions [13].

Broad strategies are needed to address this situation, including evolving the screening strategies for DR by leveraging technologies such as telemedicine and artificial intelligence (AI). Screening for DR detection is crucial, because early treatments mean better outcomes [14]. The advantages of AI screening systems include the convenience of point-of-care access and potentially lower operating costs due to automatic interpretations of images and referral to eye care specialists as needed [15].

Several studies have focused on DR grading using retinal images objectively evaluated by different AI-based grading systems [16]. One of the earliest works on DR grading from retinal fundus photographs with neural networks is by Gulshan et al. [17]. They trained a deep convolutional neural network, a specific neural network for digital image analysis, to correctly classify the disease from an aggregate pixels’ analysis. Their mathematical function does not explicitly recognize lesions like MAs and HEMOs. It learns how to identify them using local features. The neural network used in their work is described elsewhere [18]. Yun et al. [19] proposed a method to classify the DR stages using a three-layer feedforward neural network based on blood vessel examination and differentiation from MA and HEMO. Imani et al. [20] tested a new technique based on a morphological component analysis algorithm to discriminate between normal and pathological retinal structures, spotting blood vessels and exudation. Other research focused on the localization of DR traits, such as red lesions and exudates, intending to grade the severity of the disease properly. Tariq et al. [13] created a system that extracts the macula and optic disc locations from digital retinal images alongside possible exudate regions. They used a Gaussian mixture model classifier at different stages of diabetic maculopathy. Monemian and Rabbani [21] formulated a new red lesions extraction method to determine the disease’s severity better. They found the boundary pixels of blood vessels and red lesions by analyzing directional intensity changes. Sun et al. [22] proposed a new deep learning model for DR detection based on super-resolution performed on low-quality fundus images to address the issue of low image resolution, the tiny size of MAs, and insufficient imaging depth. They used a magnified adaptive feature pyramid network and a standard two-stage detection network as the backbone.

All the methods presented above, both for classification and detection, require a rigorous and deep background knowledge of programming and algorithm development, which is not an ordinary skill among healthcare professionals. Several companies have released application programming interfaces (APIs) and online interfaces to solve this issue, implementing deep learning, so that anyone with basic computer competence could train a high-quality model [23].

Our work aimed to develop a computer-assisted method that could accurately detect DR lesions from retinal fundus photographs, even without coding experience.

The contributions of this study are as follows:

- We create a dataset of annotated fundus retinal images from [24] that can be used for machine learning (ML) and computer vision purposes;
- We test the new computer vision model YOLOv9 with publicly available retinal fundus images from a well-recognized online database [24,25];
- We explore the feasibility of automated ML model development without coding expertise;
- We investigate the model’s performance in detecting different DR features, such as MAs, HEMOs, and EXs, and compare the performance to our previous research experience and other models published in the literature.

The study is structured as follows: Section 2 presents related work with use of the YOLO architecture and briefly describes our previous academic experience with YOLOv8. Section 3 outlines the proposed methodology, dataset preparation, and describes the characteristics of YOLOv8 and v9. The findings are presented in Section 4. Section 5 discusses this study’s contributions and concludes it.

## 2. Related Work

You Only Look Once (YOLO) is a state-of-the-art AI architecture by Ultralytics for training and deploying accurate AI models [26]. YOLO is an efficient object recognition algorithm characterized by its ability to perform object localization and classification in a single forward pass [27]. Version 8 sets new standards in real-time detection and segmentation, with increased different-sizes object detection capabilities and a powerful identification efficiency compared to the previous version, and it is accessible even online [28,29].

Many researchers have used the YOLO network for object detection rather than other network frameworks because of its faster speed and higher accuracy. Park et al. [30] used YOLOv3 to find the location of the optic disc (OD) and its vertical cup-to-disc ratio. Li et al. [31] tested a series of state-of-the-art algorithms for DR lesion detection, including YOLO, achieving only poor performances, suggesting that the detection task in fundus images is demanding and challenging. Santos et al. [32] proposed considerable work presenting a convolutional neural network structure based on the architectures of YOLO versions 4 and 5 to improve fundus lesion detection. They tried to keep the computational costs as low as possible, performing real-time inferences on affordable GPU devices. They improved the pre-processing phase to enhance feature extraction and minimize false positive pixel generation. They focused on creating a tiling method to increase the receptive fields of the input images, reducing the loss of information caused by the reduction in the images used at the network entrance, especially in the case of small objects such as MAs.

Our research, which tested the performance of YOLOv8 for academic use, has opened up exciting possibilities for further exploration. We developed a Bachelor’s degree thesis on DR lesion detection with the help of our optometry student. The student manually labelled a series of retinal fundus photographs with the help of a VGG image annotator, highlighting the OD, MAs, and HEMOs. They prepared four different versions of the datasets using the YOLO API service, applying different stages of data augmentation. The student compared the performances of the five available YOLOv8 models in detecting the above features through mean average precision (mAP) [33]. We also performed further experiments with the YOLOv8 online platform (image annotator, dataset preparation, data augmentation, and detection) to accurately segment and detect the OD, MAs, and HEMOs [34,35]. Our results were promising, boosting our enthusiasm to continue our research, improve it, explore future outcomes, and better understand the limitations we found. Table 1 lists the above research papers and summarizes their findings.

## 3. Materials and Methods

In this work, we focus on the detection performances on MAs, HEMOs, and EXs with the help of YOLOv9. We also describe our experience with YOLOv8 in detecting ODs, MAs, and HEMOs and compare the two model versions. MA detection is one of the most challenging tasks, as discussed by [32]; YOLOv8 can successfully detect ODs. We want to improve MA and HEMO detection by adding EX localization with the help of YOLO’s new version 9.

The experiments were carried out using an Intel Core i7, 64 GB of RAM, and an 8 GB graphic card, NVIDIA 3070Ti.

### 3.1. Dataset

First, we collected retinal fundus photographs from DR patients. We looked for available image datasets online for healthcare research purposes and found the Messidor dataset [24]. The Messidor contains 1200 eye fundus RGB color numerical images of the posterior pole, 540 healthy images and 660 DR images. The Messidor project produced this dataset to evaluate different lesion segmentation methods for color fundus images in the DR screening and diagnosis framework. It provides 8 bits per color plane image at 1440 × 960, 2240 × 1488, or 2304 × 1536 pixels, eight hundred images with pupil dilation and four hundred without pupil dilation. Three ophthalmologic departments took these pictures using a color video 3CCD camera on a Topcon TRC NW6 non-mydriatic retinographer with a 45° field of view. Each department provided 100 images in TIFF format and an Excel file with medical diagnoses. We corrected potential sources of errors in the dataset at the beginning of this research using the additional material supplied on the Messidor website. We deleted image duplicates and adjusted inconsistent grades [36].

Medical experts provided each image’s DR grade and macular edema risk. They graded the DR severity into four levels based on MA, HEMO, and neovascularization (NV) presence and amount. They graded the macular edema risk according to hard exudates. The grading system for DR and ME is shown in Table 2.

We meticulously selected 100 DR fundus RGB images from the Messidor dataset to annotate the desired features. The selected images, all of which are gradable, had varying DR severity from 2 to 3 according to the reference scale reported in Table 2. We annotated each image with pixel-level and bounding-box annotations (see section below for detailed description).

### 3.2. Annotation Method

The Messidor dataset provides image-level DR and ME grading annotations using an Excel file, referring to a number per image according to its reference scale, as shown above. It does not provide pixel-level annotations or bounding-box annotations of lesions associated with DR.

**Figure 1:**
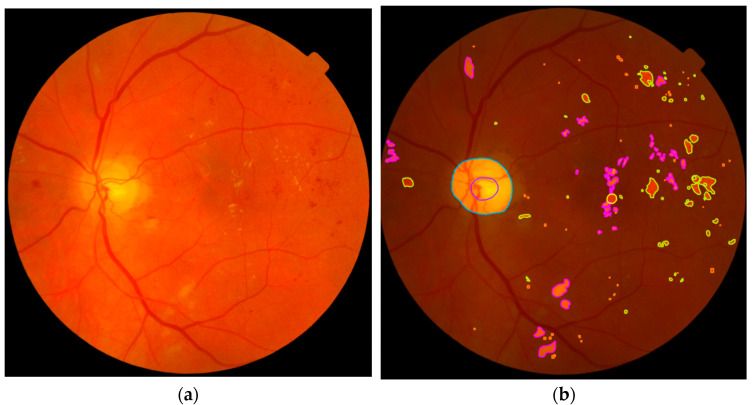

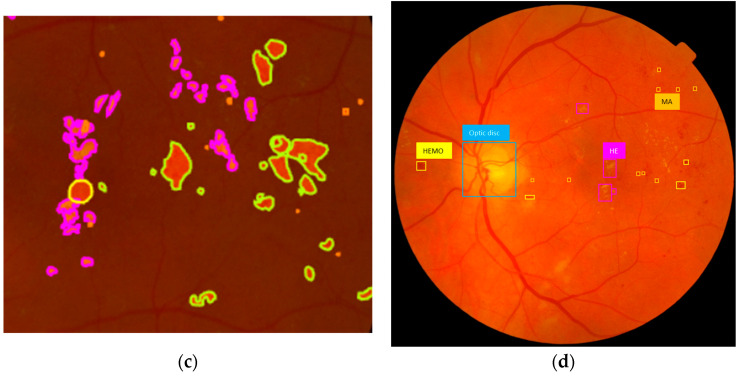
Example of (**a**) original image; (**b**) pixel-level annotated image; and (**c**) bounding-box-level annotated image: yellow, hemorrhages; cyan, optic disc; purple, exudates; and orange, micro-aneurysms. The image in (**d**) does not contain every bounding box, but just a few of them, to avoid confusion and allow better visualization.

The annotation process was a meticulous task performed by a well-trained optometrist. Using special online software provided at [37], the optometrist performed pixel-level annotations for the OD and three lesions, including MAs, HEMOs, and EXs. The annotation tool allowed the optometrist to label each lesion with a custom polygon manually, enabling them to identify boundaries and correct the polygon to follow each object profile better. The selected 100 DR RGB images were then converted into PNG format and uploaded on the YOLO API service, and the annotation process began. Each image was manually annotated, saved, and stored online for later use or downloading. We used four colors to distinguish four features. Finally, the software automatically created a copy of the pictures with the bounding-box-level annotations from the pixel-level annotated images by overlying the bounding boxes on the original image. Figure 1 provides an example of the original image and the same picture with the pixel-level and bounding-box-level annotations.

### 3.3. You Only Look Once (Version 8 and 9) Architecture

**Figure 2:**
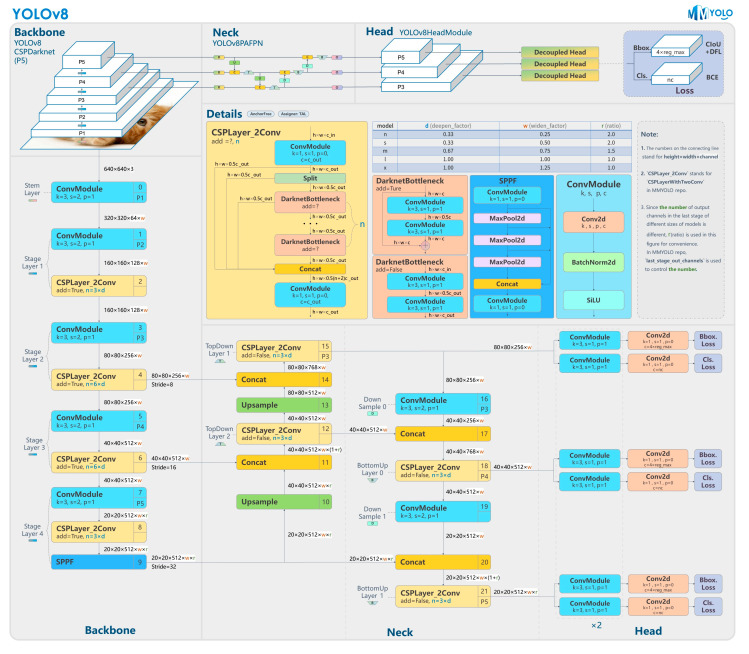
YOLOv8 architecture from [41]. It uses a modified CSPDarknet53 backbone. The C2f module replaces the CSPLayer of YOLOv5. A spatial pyramid pooling fast (SPPF) layer increases speed computation by pooling features into a fixed-size map. The head is decoupled to independently process objectness, classification, and regression tasks [38].

YOLOv8, a cutting-edge convolutional neural network (CNN) model for object detection, offers a promising blend of speed and accuracy. It addresses the problem of detecting multiple eye signs and characterizing DR as a segmentation task, facilitating the identification of different stages or severity levels of the disease. The network architecture has three main components: the backbone, the neck, and the head, as shown in Figure 2. YOLOv8, while sharing a similar backbone with YOLOv5, introduces innovative changes in the cross-stage partial connections (CSP) layer, now known as the C2f module. This module, a cross-stage partial bottleneck with two convolutions, effectively merges high-level features with contextual information, thereby enhancing detection accuracy [38]. YOLOv8 adopts an anchor-free model with a decoupled head, allowing for the independent processing of objectness, classification, and regression tasks. This design, which enables each branch to concentrate on its specific task, significantly enhances the model’s overall accuracy. In the output layer of YOLOv8, the sigmoid function is used as the activation function for the objectness score, indicating the likelihood that the bounding box contains an object. The softmax function is employed for the class probabilities, indicating the likelihood of the objects belonging to each possible class [38]. YOLOv8 leverages the CIoU [39] and DFL [40] loss functions for bounding-box loss and binary cross-entropy for classification loss. These losses significantly enhance object detection performance, particularly when dealing with smaller objects, instilling confidence in the model’s capabilities [38].

The backbone [42] is responsible for extracting rich feature representations from the input image *I*, which is defined as:

(1) $$I\epsilon R^{H\times W\times 3},$$

where *H* and *W* are the height and width of the input image, respectively. A series of convolutional layers are applied to the input image to extract features.

(2) $$Conv\left(I,\;K,\;s,\;p\right)=ReLU(BatchNorm\left(I*K\right)),$$

where *K* is the convolutional kernel, *s* is the stride, *p* is the padding, * denotes the convolution operation, and BatchNorm denotes the batch normalization. Residual blocks help to learn deeper features:

(3) $$Res\left(X\right)=X+Conv(Conv\left(X,K_{1},\;s_{1},\;p_{1}\right),K_{2},\;s_{2},\;p_{2}),$$

where *X* is the input of the residual block, and *K*_1_ and *K*_2_ are the kernels of the convolutional layers within the block.

The neck [42] aggregates feature maps from different stages of the backbone to enhance feature representation. The Feature Pyramid Network (FPN) combines feature maps from different scales:

(4) $$P_{l}=UpSample\left(C_{l+1}\right)+C_{l},$$

where *P_l_* is the feature map at level *l*, *C_l_*_+1_ is the feature map from the previous layer, and UpSample denotes the up-sampling operation. The Path Aggregation Network (PAN) enhances the feature pyramid by combining feature maps in both top–down and bottom–up pathways:

(5) $$U_{l}=Conv(ConcatP_{l-1},DownSample\left(P_{l}\right))),$$

where *U_l_* is the output feature map at level *l* and Concat denotes the concatenation operation.

The head [42] predicts the bounding boxes, objectness scores, and class probabilities for the detected objects. The bounding box prediction is defined as:

(6) $$B=(\sigma \left(t_{x}\right)+c_{x},\sigma \left(t_{y}\right)+c_{y},e^{t_{w}}\cdot p_{w},e^{t_{h}}\cdot p_{h})\;,$$

where (*c_x_*, *c_y_*) is the center of the anchor box, (*p_w_*, *p_h_*) are the dimensions of the anchor box, and *t_x_*, *t_y_*, *t_w_*, and *t_h_* are the predicted offsets. The sigmoid function *σ* ensures that the outputs are within a valid range. The objectness score is defined as:

(7) $$o=\sigma (t_{c}),$$

where *t_c_* is the raw class score for class *c*.

The loss function used to train YOLOv8 combines multiple components to ensure accurate predictions. The localization loss is defined as:

(8) $$l_{loc}=\sum_{i}{smooth}_{L1}(B_{i},{B^{\prime }}_{i}),$$

where *B_i_* is the predicted bounding box, *B*′*_i_* is the ground truth bounding box, and *smooth_L_*_1_ is the smooth *L*1 loss. The objectness loss is defined as:

(9) $$l_{obj}=\sum_{i}BCE(o_{i},{o^{\prime }}_{i}),$$

where *BCE* is the binary cross-entropy loss, *o_i_* is the predicted objectness score, and *o*′*_i_* is the ground truth objectness score. The classification loss is defined as:

(10) $$l_{cls}=\sum_{i}BCE(p_{i}(c),{p^{\prime }}_{i}(c)),$$

where *p_i_* (*c*) is the predicted class probability and *p*′*_i_* (*c*) is the ground truth class label. The total loss is then defined as:

(11) $$l=\lambda_{loc}l_{loc}+\lambda_{obj}l_{obj}+\lambda_{cls}l_{cls},$$

where *λ_loc_*, *λ_obj_*, and *λ_cls_* are weighting factors for each loss component [42].

**Figure 3:**
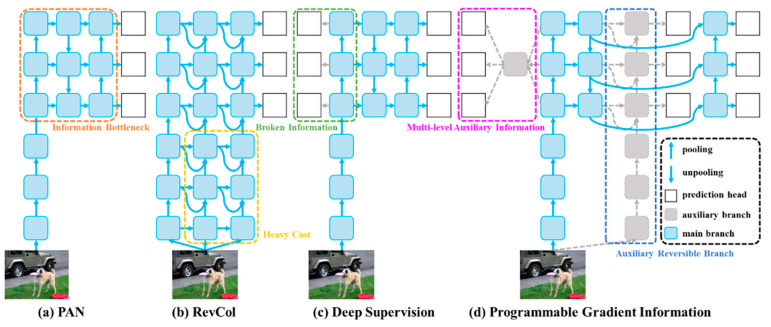
YOLOv9 architecture from [25]. (**a**) Path Aggregation Network (PAN) [43], (**b**) Reversible Columns (RevCol) [44], (**c**) conventional deep supervision, and (**d**) Programmable Gradient Information (PGI). The PGI is formed by three main components: main branch, auxiliary branch, and multi-level auxiliary information.

YOLOv9 marks a significant advancement in real-time object detection, introducing groundbreaking techniques such as Programmable Gradient Information (PGI) and the Generalized Efficient Layer Aggregation Network (GELAN) [25]. The new version, developed upon the robust codebase of YOLO version 7, shows remarkable efficiency, accuracy and adaptability improvements. Information loss in deep neural networks is a critical challenge that YOLOv9’s advancements try to address. The core innovations of version 9 lay in the Information Bottleneck Principle (IBP) and Reversible Functions (RFs). Figure 3 shows the architecture diagram of YOLOv9.

The IBP highlights a crucial challenge in deep learning: as data pass through multiple layers of a network, the information loss increases. This phenomenon is mathematically represented as:

(12) $$I(X,\;X)\geq I(X,\;f_{\theta }\left(X\right))\geq I(X,g_{\varphi }\left(f_{\theta }(X\right))),$$

where *I* means mutual information and *f* and *g* are transformation functions with parameters *θ* and *φ*, respectively. This loss can lead to unreliable gradients and a poor model convergence. One solution is to increase the model’s size, retaining more information. YOLOv9 counters this challenge by implementing PGI, which aids in preserving essential data across the network’s depth, ensuring more reliable gradient generation, convergence, and performance. PGI is a solution comprising a main branch for inference, an auxiliary reversible branch for reliable gradient calculation, and multi-level auxiliary information to tackle deep supervision issues effectively without adding extra inference costs.

A function is defined as reversible if it can be inverted without any loss of information, as expressed by:

(13) $$X=v_{\zeta }(r_{\psi }(X)),$$

where *ψ* and *ζ* are parameters for the reversible and its inverse function. This ensures no information loss during data transformation, enabling the network to maintain all the input data through all the layers and provide more accurate updates to the model’s parameters. YOLOv9 incorporates RFs within its architecture to mitigate the risk of data degradation and preserve critical information for object detection tasks.

GELAN represents a unique design that fits the PGI framework, enabling YOLOv9 to achieve superior parameter utilization and computational efficiency. It allows for the flexible integration of various computational blocks, making version 9 adaptable to various applications without sacrificing speed or accuracy. For more detailed information on YOLOv9 and the YOLO family, see [25,45].

Table 3 shows the available variants of YOLO versions 8 and 9, which are accessible online for project development, highlighting the sizes of input images (in pixels), number of used parameters (in millions), and floating-point operations per second (FLOPs, i.e., number of parameter and computational needs). YOLOv8 comes in nano (n), small (s), medium (m), large (l), and extra-large (x) model sizes. YOLOv9 offers model variants from tiny (t) to small, medium, compact (c), and extensive (e). We used the c and e versions of YOLOv9 for this research, the only two available iterations at the beginning of this work [45,46].

### 3.4. Data Augmentation and Training Parameters

Before we started the training phase, we increased our input dataset of 100 images by applying data augmentation to increase the dataset diversity and avoid overfitting. We performed image resizing to a resolution of 640 × 640 pixels, set the auto-orientation function, and applied a 4 rows × 4 columns tiling. Horizontal and vertical flipping, 90° rotations clockwise, counter-clockwise, and upside down were applied. We created a 4256-image dataset, with 94% and 6% of the images used for training and validation. Batch size, a hyperparameter defining the number of samples taken to work through a particular machine learning model before updating its internal model parameters, was set at 8. The default value is 32, but we decided to try smaller batch sizes first, because a more significant size typically requires a lot of computational resources to complete an epoch, but requires fewer epochs to converge. We opted to run each model for 100 epochs and set an early-stop parameter, monitoring the progress of the training phase. If the model did not improve for ten consecutive epochs, it stopped, as it had converged.

### 3.5. Performance Metrics

The performance metrics [32,47] used to analyze the research outcomes include:

- Average Precision (AP): AP computes the area under the Precision × Recall curve, providing a single value that encapsulates the model’s precision and recall performance;
- Mean Average Precision (mAP): this extends the concept of AP by calculating the average AP values across multiple object classes, as shown in Equation (14). It immediately provides a comprehensive evaluation of the model’s performance. It is commonly used in computer vision model research to compare both different models on the same task and different versions of the same model;
- Precision (P) and Recall (R): the first quantifies the proportion of true positives among all positive predictions, assessing the model’s capability to avoid false positives. The latter calculates the proportion of true positives among all actual positives, measuring the model’s ability to detect all instances of a class. Precision and Recall are calculated for each class by applying the formulas for each image, as shown in Equations (15) and (16), respectively;
- Accuracy (Acc): Acc is a metric that measures how often a model correctly predicts the outcome. In other words, accuracy is equal to the number of correct predictions divided by the number of predictions made, as shown in Equation (17);
- F1 score: this the harmonic mean of Precision and Recall, providing a balanced assessment of a model’s performance while considering both false positives and false negatives, as shown in Equation (18);
- Intersection over Union (IoU): this is used to estimate the similarity between two sets of samples, and the ratio between the area of overlap and the area of the union of the predicted bounding boxes and the ground truth bounding boxes obtains it.

(14) $$mAP=\frac{1}{N}\sum_{i=1}^{N}{AP}_{i},$$

(15) $$Precision=\frac{TP}{TP+FP},$$

(16) $$Recall=\frac{TP}{TP+FN},$$

(17) $$Accuracy=\frac{TP+TN}{TP+TN+FP+FN},$$

(18) $$F1\;score=\frac{TP}{TP+\frac{1}{2}(FP+FN)},$$

where *TP* is true positive, *TN* is true negative, *FP* is false positive, and *FN* is false negative.

**Figure 4:**
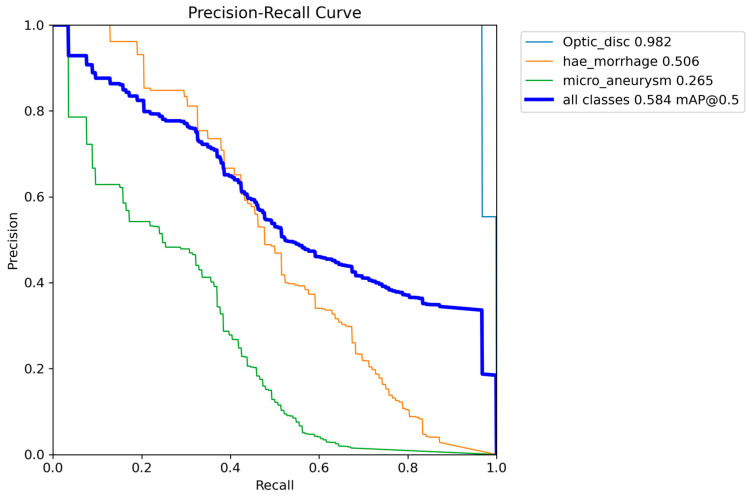
Graph with Precision × Recall curve with a limit of IoU of 0.5 obtained during the validation step of the proposed approach with YOLOv8 model-*s*.

We provide a Precision–Recall curve example, a useful tool in model performance evaluation. From this curve, we can calculate AP and mAP as the weighted means of the Precisions achieved at each threshold, with the increase in Recall from the previous threshold used as the weight. An IoU of 0.5 is selected to calculate the proposed model’s performance and compare the results with the other works from the literature. Figure 4 shows the PR curves from the YOLOv8 model-*s* used to detect ODs, MAs, and HEMOs. The *x*-axis of the PR curve represents the Recall, while the *y*-axis shows the Precision. In this space, the goal is to be in the upper right corner (1, 1), meaning that the predictor classified all positives as positive (Recall = 1), and that everything classified as positive is true positive (Precision = 1) [32]. We use the top right corner summary table to identify the performance achieved by the model on each class, showing the AP per class and the calculated mAP at an IoU of 0.5.

**Figure 5:**
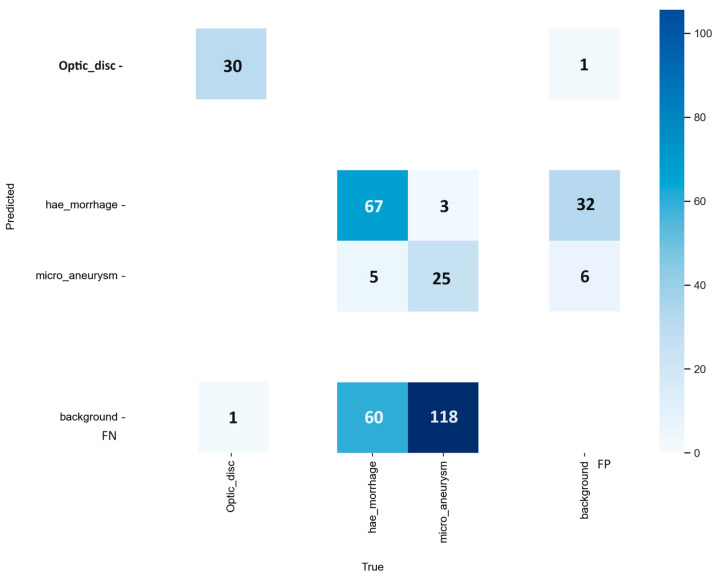
Confusion matrix from the model-*s* of YOLOv8 experiment, during the validation step of the proposed approach. Optic discs; hemorrhages; and microaneurysms. Background, *x*-axis represents the false positive (FP), *y*-axis stands for false negative (FN).

Figure 5 shows an example of a confusion matrix, a table containing data from experiments with the adopted approach. It summarizes the information on the performances achieved and lets us compare them to other work. For example, we show the confusion matrix from the same experiment with the YOLOv8 model-*s* as before. To better understand how to use a confusion matrix, we use Equations (15) and (16) to calculate the Precision and Recall from the confusion matrix for the OD class. The computation is available below.

The confusion matrix resulting from the detection of objects presents the numbers of false positives (FPs) and false negatives (FNs), respectively, the image background detected as a lesion, without any corresponding label in the ground truth, and authentic objects not detected by the proposed method and, therefore, considered as background. True positives (TPs) and true negatives (TNs) are found in the confusion matrix as well, respectively, a lesion with a corresponding label in the ground truth detected as an effective lesion by the model, and a result that indicates the absence of a lesion or a feature. The confidence limit established for detecting objects in these images will directly impact the results obtained from background FPs and background FNs. A confidence limit is applied to filter the bounding boxes of a possible object to eliminate the bounding boxes with low confidence scores through a Non-Maximum Suppression algorithm, which disregards detected objects with an IoU less than the defined threshold [32]. We calculated the results presented in the confusion matrix using a fixed confidence limit of 0.25, which aligns with the default inference configurations of YOLOv8 and v9. With lower confidence limits, such as our default value, the mAP results will be improved but produce a more significant amount of background FPs that will appear in the confusion matrix [32]. Squares with darker shades of blue indicate a more significant number of samples. The confusion matrix presents the hits in predicting fundus lesions on the main diagonal, while the values off the main diagonal correspond to prediction errors.

We show and compare the results using only the variants of the models with the highest mAPs, choosing between *n*, *s*, *m*, *l*, and *xl* options for YOLOv8 and between *c* and *e* for YOLOv9. For each selected variant, we calculate and provide the Precision, Recall, accuracy, and F1 score.

To calculate the Precision and Recall for the OD class, according to Equations (15) and (16), we need TPs, FPs, and FNs for the OD class. As we can see from the confusion matrix in Figure 5, the box connecting the true Optic_disc on the *x*-axis and the predicted Optic_disc on the *y*-axis contains the number 30. This number represents the TPs for the OD class. Similarly, if we want to find the FPs and FNs for the OD class, we have to look at the boxes which connect the background on the *x*-axis to the Optic_disc on the *y*-axis and the Optic_disc on the *x*-axis to the background on the *y*-axis, respectively. Then, the FPs and FNs for the OD class are equal to 1. Now, applying Equations (15) and (16), the Precision and Recall are equal to 0.968. If we calculate the same performance metrics for another class, such as MA, the TPs are 25, the FPs are 6, and the FNs are 118. The Precision and Recall for MA are 0.806 and 0.175, respectively. Repeating this procedure, it is possible to calculate performance metrics for all the desired classes.

## 4. Results

We successfully built a digital image dataset with DR lesions (MAs, HEMOs, and EXs) and OD annotations from the Messidor database. We ran the same dataset on two different versions of the YOLO architecture, YOLOv8 and YOLOv9, and compared the feature detection ability results. We first describe OD, MA, and HEMO segmentation with YOLOv8 and then present the novel results from the YOLOv9 segmentation of MAs, HEMOs, and EXs.

We carefully evaluated our proposed approach using the AP and mAP metrics, ensuring a comprehensive comparison of the results. These metrics, which measure the precision of machine learning algorithms in object detection, were instrumental in our evaluation. We then compared our the YOLO v8 and v9 results with related approaches found in the literature, further validating our findings.

### 4.1. OD, MA, and HEMO with YOLOv8

The small version of the model performed best in detecting ODs, MAs, and HEMOs with the highest mAP. Figure 4 visually presents the PR curves for the small model. The APs for ODs, MAs, and HEMOs are 0.982, 0.265, and 0.506, respectively. The mAP for all fundus features considered is 0.584 at an IoU of 0.5, underscoring the small model’s superior performance. Figure 5 depicts the *s*-variant’s confusion matrix, offering a detailed breakdown of the model’s performance. The matrix reveals that the highest incidence of background FNs occurred in MAs (with 82%), followed by HEMOs (with 36%) and ODs (with 3%). In terms of FP background errors, the highest incidence was in HEMOs (with 67%), followed by MAs (with 10%) and ODs (with 1%). The system also incorrectly identified 7% of HEMOs as MAs and 3% of MAs as HEMOs, highlighting areas for potential improvement.

All the performance results obtained by each model variant are shown in Table 4, with the metrics of AP and mAP for the IoU threshold of 0.5 in the validation dataset.

**Figure 6:**
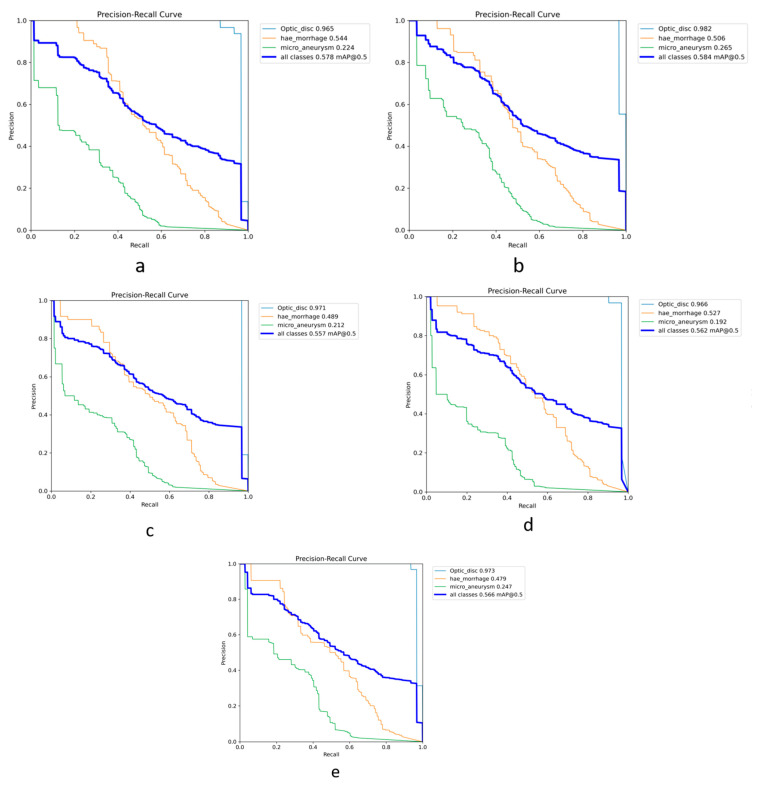
All PR curves from each model’s size of YOLOv8 experiment on OD, MA, and HEMO detection, with classes’ AP and mAP. Variants are shown as follows: (**a**) nano, (**b**) small, (**c**) medium, (**d**) large, and (**e**) extra-large. Note how visually similar curves are, making accurate interpretation difficult.

We opted to display all the model variant results in a tabular form, leveraging their visual similarity. This method allows the reader to swiftly pinpoint the best model variant, as only its results are shown graphically, as shown in Figure 4. To further substantiate our decision, we present an example in Figure 6, displaying the results from the five model sizes of YOLOv8 when ODs, MAs, and HEMOs are considered.

The OD detection is higher than 96% with all variants of YOLOv8, indicating that this hallmark of the posterior pole of the eye can be accurately highlighted with this online API CNN, with no need for external modification by coding experts. The adaptability of the YOLOv8 variants ensures their versatility in various applications. Moreover, this task can be efficiently and accurately fulfilled with the reduced size *s*-variant of the model, keeping the computation load at a minimum and the operation time as low as less than ten minutes. MA detection is highest with the same *s*-version, followed by the *xl*-version. HEMOs, instead, are best detected with the nano version of the model, followed by the large and then small variants.

After this experiment, we remove the MA class from the dataset and try to rerun each model to see if there is room for improvement in HEMO detection. We leave the OD class to test if the CNN consistently repeats this task, confirming an excellent capability to segment the OD and HEMOs. Again, the best-performing version of the model is the small one, with an mAP of 0.769 at an IoU of 0.5. The APs for OD sand HEMOs are 0.982 and 0.555, respectively. All the performance results obtained by each variant of the model are shown in Table 5, with the metrics of AP and mAP for the IoU threshold of 0.5 in the validation dataset. OD segmentation has the best detection AP, with the medium version of the model performing slightly better than the small one, followed by the *xl*, *n*, and *l* variants. Version *n* and *m* perform equally in HEMO segmentation, followed by *xl* and *l*, confirming the *s*-model as the best.

**Figure 7:**
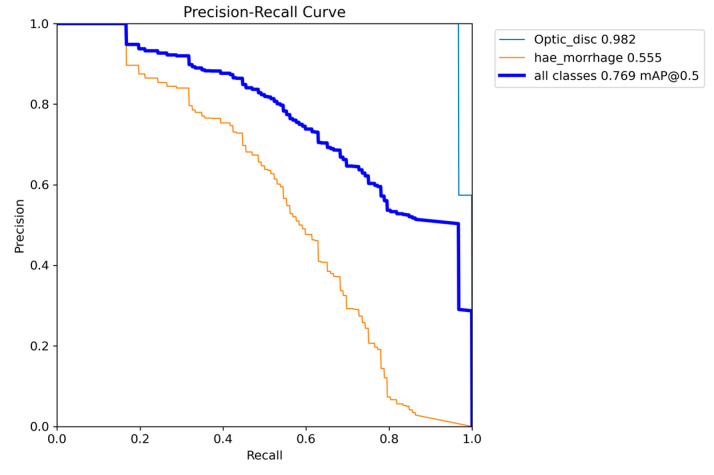
Graph with Precision × Recall curve with a limit of IoU of 0.5 obtained during the validation step of the proposed approach with YOLOv8 model-*s*, excluding the MA class from the analysis.

**Figure 8:**
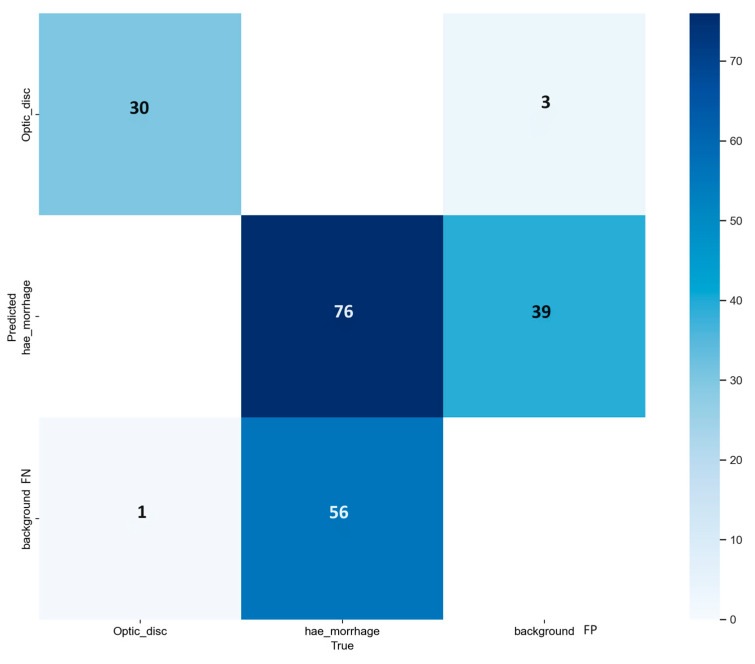
Confusion matrix from the model-*s* of YOLOv8 experiment, during the validation step of the proposed approach, excluding the Microaneurysms class. Background, *x*-axis represents the false positive (FP) and *y*-axis stands for false negative (FN).

Figure 7 shows the PR curves from this modified model, where the curves are closer to the top right corner, indicating finer lesion detection. Remarkably, the curve in orange, which is from HEMO segmentation, is more curved towards the top right corner than the one from Figure 4. Deleting the MA class from the training phase makes the model a better classifier, with the mAP increasing from 0.506 to 0.555. The blue mAP curve draws a similar pattern, with a better curved profile in Figure 7 than in Figure 4, meaning that the new *s*-model has better segmentation skills. Figure 8 shows its confusion matrix: the HEMO class reached background FN and FP rates of 42% and 93%. There was no confusion between ODs and HEMOs during detection, probably thanks to the OD’s more prominent size and higher contrast against the color fundus of the eye. The edges of red lesions, such as HEMOs and even more MAs, can be covered by the color of surrounding pixels, similar to the posterior pole pictures.

### 4.2. MA, HEMO, and EX with YOLOv8

**Figure 9:**
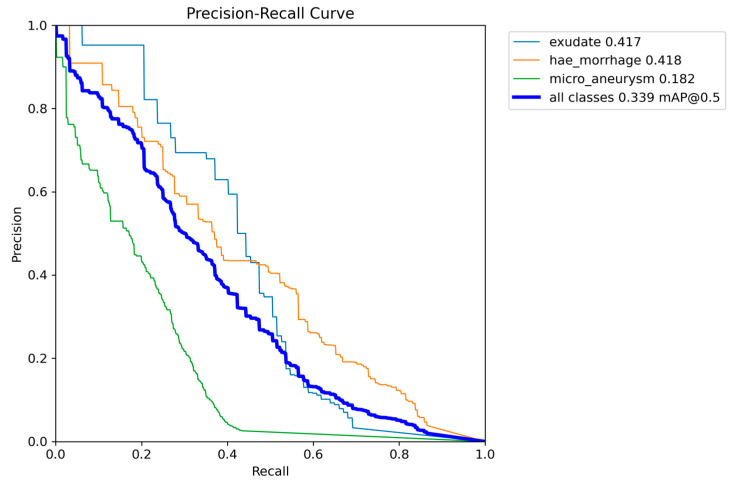
Graph with Precision × Recall curve with a limit of IoU of 0.5 obtained during the validation step of the proposed approach with YOLOv8 model-*n.*

**Figure 10:**
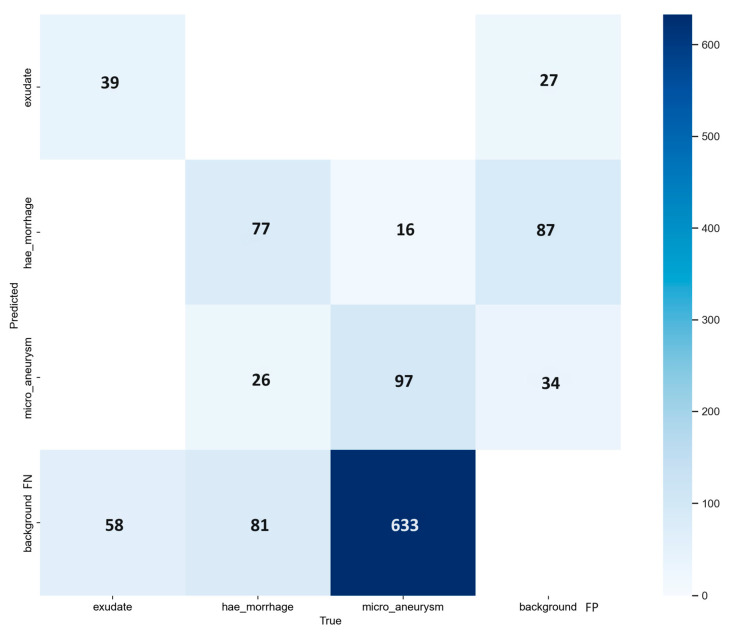
Confusion matrix from the model-*n* of YOLOv8 experiment, during the validation step of the proposed approach; EX, HEMO, and MA classes. Background, *x*-axis represents the false positive (FP) and *y*-axis stands for false negative (FN).

As we successfully achieved OD segmentation with the first couple of experiments, we tested YOLOv8 in detecting MAs and HEMOs again, adding a white lesion class as EXs. We achieved the best mAP with two versions of the model, the *n* and *m*-models, with a value of 0.339 at IoU 0.5. We chose the nano version because of its faster training time, lower computation load, and higher AP registered for the EX class (0.417). The MA detection was very close between the two models, with the nano and medium reaching APs of 0.182 and 0.189, respectively. The *m*-model better segmented HEMOs, reaching an AP of 0.483. We still decided to choose the nano because, in the previous experiment, we achieved a better performance both for the MA and HEMO classes, so we chose the variant which gave the best outcome for the new EX class. While performing the training phase with the *xl*-model, we faced several system aborts, leading to a loss of trained data and the absence of performance metrics for this variant. All the results are shown in Table 6, with the metrics of AP and mAP for the IoU threshold of 0.5 in the validation dataset. Figure 9 shows the PR curves from the *n*-model, which depicts the AP and mAP for each class, and Figure 10 represents its confusion matrix. The highest incidence of background FNs occurred in MAs (with 85%), followed by EXs (with 60%) and HEMOs (with 44%). As for FP background errors, the highest incidence occurred in HEMOs (with 59%), followed by MAs (with 23%) and EXs (with 18%). Finally, the system misclassified 14% of HEMOs as MAs and just 2% of MAs as HEMOs.

### 4.3. MA, HEMO, and EX with YOLOv9

**Figure 11:**
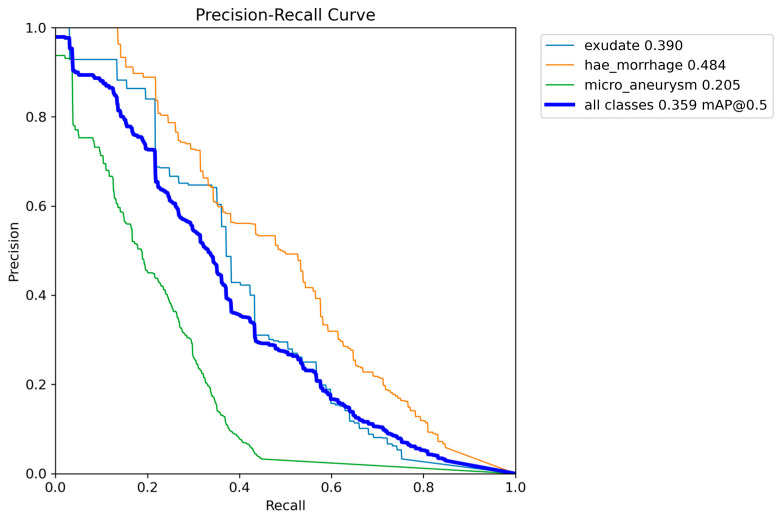
Graph with Precision × Recall curve with a limit of IoU of 0.5 obtained during the validation step of the proposed approach with YOLOv9 model-*e.*

**Figure 12:**
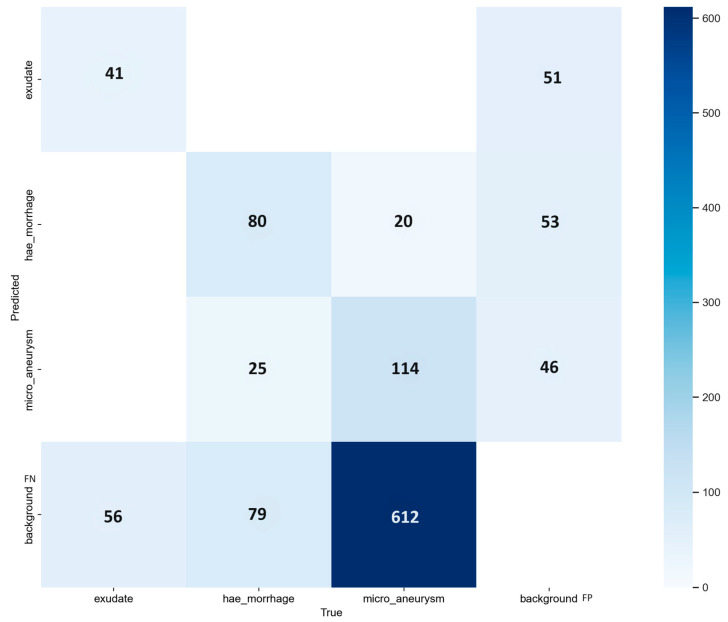
Confusion matrix from the model-*e* of YOLOv9 experiment, during the validation step of the proposed approach; EX, HEMO and MA classes. Background, *x*-axis represents the false positive (FP) and *y*-axis stands for false negative (FN).

We performed the last experiment with the two models offered by YOLOv9, the *c* and *e*-models. We trained the two options with the same dataset as the previous experiments and evaluated the MA, HEMO, and EX detection performance. The best result was achieved by the *e*-model, with an mAP of 0.359 at an IoU of 0.5. AP for MAs, HEMOs, and EX, with values of 0.205, 0.484, and 0.390, respectively. The compact version of the model showed an almost identical mAP of 0.358, with a slightly improved AP for MAs at 0.212, the same AP for HEMOs as the extended variant, and a lower EXs AP value of 0.378. All the results are shown in Table 7. Figure 11 and Figure 12 show the PR curves and confusion matrix for the extended model. The most significant number of background FNs occurred in MAs (with 82%), followed by EXs (with 58%) and HEMOs (with 43%). As for FP background errors, the highest incidence occurred in HEMOs (with 35%), followed by EXs (with 34%) and MAs (with 31%). Ultimately, the model incorrectly detected 14% of HEMOs as MAs. Conversely, it mistook just 3% of MAs for HEMOs.

### 4.4. Summary of Experiment Results

We describe the results from four experiments, three with YOLO version 8 and one with version 9. We evaluated the performance of four computer vision models in detecting ODs, MAs, HEMOs, and EXs with metrics such as mAP and AP, and then with the FPs and FNs from their confusion matrixes. Table 4, Table 5, Table 6 and Table 7 show our findings in detail and compare the various options available within the YOLOv8 and v9 networks. Considering the confusion matrixes from Figure 5, Figure 8, Figure 10, and Figure 12, we can calculate the FPs and FNs for each of the best models from every experiment. In medical imaging analysis, the role of FNs is crucial, because the cost of a high FN rate could mean potentially misdiagnosed life-threatening conditions, or in our case, eye-threatening conditions. It is possible to verify that every time there is an MA detection task, which involves identifying microaneurysms, MAs reach the highest incidence of FNs (82% and 85%), followed by EXs (58% and 60%), HEMOs (36%, 42%, and 44%), and ODs (3%). A higher rate of FN could mean the systems struggle to accurately detect a lesion, leading to a possible misdiagnosis. MA detection is the most complex task in this kind of computer vision experiment [32], and our experiments confirmed this belief. MAs are the first and earliest sign of DR, and accurate, objective localization of these lesions is tremendously desirable in large population-based screening and clinical practice for better prevention and treatment outcomes. Future works must prioritize the detection of early signs of disease with larger datasets such as [29,48] with a clinically acceptable performance compared to medical retina experts.

Table 8 summarizes the performance metrics of mAP, AP, Precision, Recall, Accuracy, and F1 score achieved by each of the selected models from our experiments with YOLOv8 and v9.

Precision considers how many predictions are correct among all the lesion-positive assumptions made by the model; Recall assumes how many situations of the positive class with expected values are correct; the F1 score calculates the harmonic mean between P and Re, giving a more balanced evaluation of the model’s accuracy. The first experiment is 50% accurate, with an F1 score of 0.528, which is acceptable. The lower Re value of 0.405 means the model could be missing actual lesions, leading to possible undiagnosed DR, especially in the early stage of the disease. When we ask the model to consider just two classes (ODs and HEMOs), the F1 score increases, and the P and Re values are more balanced (71.6%, 65%, and 68.2%, respectively). In this case, the model’s accuracy, expressed by the F1 score, is close to 70%, which means it is an excellent classifier. It is possible to consider the small version of YOLOv8 we use to detect ODs and HEMOs as a promising system to accurately and objectively detect those two hallmarks of the posterior pole when clinicians need to quickly evaluate the OD and suspect DR or any other conditions leading to bleeding of the retina. Unfortunately, the last two experiments achieve lower F1 scores, 31.6% and 34.4%, respectively. Precision is similar between the two models, but the lower Re scores lead to many missing lesions. This cause of this loss relies on MA detection, which is, again, very difficult to fulfil. The best model is the extended version of YOLOv9, which has at least all scores higher than those of the YOLOv8-nano. We need larger image datasets and improved feature extraction techniques such as [48,49] for better results.

Table 9 summarizes the performance metrics of mAP and AP achieved by each of the selected models from our experiments with YOLOv8 and v9 and related works found in the literature that provide the same evaluation metrics.

We should be satisfied with our proposed models, where the OD detection achieved an AP of 0.982. Compared to other models found in the literature, such as [30], YOLOv8 is second to DenseNet and YOLOv3 (with Aps of 1.000 and 0.990, respectively) but better than ResNet (AP 0.956). Future works with YOLOv8 and YOLOv9 will enable us to measure and evaluate the morphological characteristics of the OD, such as the vertical cup-to-disc ratio [30], which are associated with progressive disease stages in glaucoma. Similarly, researchers may use YOLO models in glaucoma detection, as they have for DR detection.

Considering the papers reporting MA, HEMO, and EX detection performance, our two models, YOLOv8-nano and YOLOv9-extended, perform well, with mAPs of 0.339 and 0.359, respectively. They seem to be better models than the options presented by [31,32,49,52] in detecting DR lesions. The DeepLabv3+ from [31] is the only neural network performing closer to our proposed approach, with an mAP of 0.332. The AP values for HEMO and EX detection with DeepLabv3+ are 0.405 and 0.438; for YOLOv8-nano, they are 0.418 and 0.417; and for YOLOv9-extended, they are 0.484 and 0.390. It is possible to verify that our proposed approaches perform better in HEMO detection. However, DeepLabv3+ is better in EX detection, probably due to the higher EX annotations in the DDR dataset.

Although the obtained AP for OD detection alone is excellent, the overall performance of the s-model of YOLOv8, as shown in Figure 4, still needs to be at the level of clinical use on actual patients. The mAP curves for OD, MA, and HEMO are not at the ideal point (1, 1) or an mAP of 1.0, nor are they at an acceptable 0.8 [55]. However, when we consider MAs, HEMOs, and EXs, as shown in Figure 9 and Figure 11 (YOLOv8 model-n and YOLOv9 model-e, respectively), the mAPs for the selected lesions are better than in other work from the literature, indicating the need for further research. The only instance where the mAP is close to 0.8, suggesting that the model could be cautiously adopted for clinical use, is shown in Figure 7 (YOLOv8 model-s). In this case, the mAP is 0.769, close to 0.8, valid for OD and HEMO detection.

The precise segmentation and detection of MAs is still a challenge in the field due to the intrinsic characteristics of these retinal lesions. Without complex image pre-processing, our approaches achieved higher results than the other experiments shown in Table 9. The nano version of YOLOv8 and the extended version of YOLOv9 reached MA APs of 0.182 and 0.205, respectively, significantly higher than the other neural networks. Even our YOLOv8-small model achieved an AP of 0.265 for MA detection, just with the unmodified version of YOLO and RGB images. The best alternatives that perform well on the DDR dataset are the two from [32], which achieved MA APs score of 0.105 and 0.111 with YOLOv8 + SGD + tilling and YOLOv8 + Adam optimizer + tilling. The best model among all the presented neural networks in Table 9 is the one from [48], which reached an AP of 0.813 in MA detection within the EyePacs image dataset.

## 5. Discussion

This work demonstrates that, even without coding expertise, optometrists, medical professionals, and researchers can achieve significant results in DR lesion detection tasks. By utilizing the training models available online as API services, private practices and public hospital teams can create image datasets and establish a platform for objectively evaluating medical images, particularly those from DR patients. YOLOv8 and v9, as powerful tools, empower these professionals to enhance patient care and healthcare services, including telemedicine and home-based examinations, while keeping costs low. This presents an excellent opportunity for students and young eye care practitioners to bolster their knowledge through education-based software created with low-cost API services, thereby enhancing their role in the diagnosis process.

YOLOv8 and v9, as excellent online systems, not only offer an annotation platform for creating desired datasets for different computer vision tasks, but also a series of working computer vision models. These models inspire innovation and progress in the field of computer-assisted diagnoses of DR, providing a platform for objectively evaluating medical images and pushing the boundaries of what is possible in healthcare.

Our creation of a 100 RGB image dataset from the Messidor database, with OD, MA, HEMO, and EX annotations, is a significant contribution to future research. While the Messidor dataset has been widely used to facilitate studies on computer-assisted diagnoses of DR, it is prone to overfitting when training with deep neural networks [32]. To ensure the quality and reliability of our research and training, we must prioritize using standardized datasets where precise descriptions of available annotations are well-defined. An example of such a dataset is the DDR [31], which provides 757 annotated DR images for both image classification, according to international DR classification standards, and lesions detection, with four pixel-level labeled classes. This dataset has been used extensively in research focused on computer-assisted lesion segmentation (see Table 9).

Using different datasets, our study compared results with other works on computer vision models and DR lesion detection. We recognize the value of mAP in computer vision research to address this issue. We plan to test the unmodified versions of YOLOv8 and YOLOv9, as in the current work, to verify their applicability in clinical contexts and by non-programming experts. Once we have gained this experience and clarified their strengths, we will be able to engage a coding expert to adjust the hyperparameters of each model for improved results, paving the way for future research directions.

Another limitation of this study is the image type used. We used RGB images and did not apply image pre-processing, except for image tiling in data augmentation. However, we are optimistic about the potential of image pre-processing, which has been adopted successfully by [48,49,56]. This process, which starts with green channel image extraction from the original RGB image, thus increasing the contrast of red lesions (they appear as black spots) and then improving the image contrast through an adaptive histogram equalization technique, holds promise for future research. Finally, it is possible to accurately separate the desired lesions from the background by applying the correct threshold and morphological operations. This procedure can be adopted even for white lesion extraction, such as exudates.

The proposed approach achieved remarkable results compared to other state-of-the-art works, with similar purposes found in the literature, confirming that machine learning techniques can successfully perform fundus lesion detection. However, this achievement needs to solve previous challenging issues, such as detecting microlesions. MA detection must still be perfected before implementation in actual practice with real patients. They are tiny and can be too close to each other, leading to frequent missing. Of course, this scenario is not acceptable. Future works should focus on new methods to properly separate MAs from the retina’s background and each other. Upscaling the image size could be a possible solution. However, this comes with a more significant computation load and the need for higher-resolution devices, which are expensive and not very widespread. However, AI is developing so fast that we will soon have a more powerful tool to detect even micro-objects with the proper input information. This will be a game changer in computer-assisted medical image classification, significantly improving patient care assistance and reducing the need for highly specialized practitioners and expensive equipment.

## Figures and Tables

**Table 1:** List of research works involving YOLO architectures.

Research Paper	YOLO Version	Main Findings
[30]	YOLOv3	OD location and its vertical cup-to-disc ratio
[31]	YOLO	MA, EX, and HE detection with poor mAP results
[32]	YOLOv4 and YOLOv5	Improved pre-processing phase to minimize false positive pixel generation; development of a tiling method to reduce the loss of information due to input image resize
[33]	YOLOv8	OD, MA, and HEMO annotation and performance comparison by mAP from five model’s sizes of YOLO
[34]	YOLOv8	OD, MA, and HEMO annotations improved; fine tuning of the dataset; performance evaluation by mAP
[35]	YOLOv8	OD and HEMO class only and performance evaluation by mAP

**Table 2:** Messidor dataset grading system. DR is classified according to the amount of MAs, HEMOs, and NV. Risk of ME is given by the number of HE.

Grade	DR Lesions	Risk of ME
0 (Normal)	No detectable lesions	No visible HE
1	Mild lesions (0 < MA ≤ 5) and no HEMO (=0)	Shortest distance between macula and HE > one papilla diameter
2	Moderate lesions (5 < MA < 15 or 0 < HEMO <5 ) and no NV (=0)	Shortest distance between macula and HE ≤ one papilla diameter
3	Severe lesions (MA ≥ 15 or HEMO ≥ 5) or presence of NV (=1)	NA

DR: diabetic retinopathy; ME: macular edema; MA: micro-aneurysm; HEMO: hemorrhages; HE: hard exudates; NV: neovascularization; and NA: not available.

**Table 3:** Comparison between YOLO version 8 and 9 model variants.

Model	Size (Pixels)	Parameters (Million)	FLOPs (B)
YOLOv8-n	640	3.4	12.6
YOLOv8-s	640	11.8	42.6
YOLOv8-m	640	27.3	110.2
YOLOv8-l	640	46.0	220.5
YOLOv8-x	640	71.8	344.1
YOLOv9-c	640	27.9	159.4
YOLOv9-e	640	60.5	248.4

**Table 4:** Results obtained by each variants of YOLOv8′s models, with the metrics AP and mAP for the IoU threshold of 0.5 in the validation dataset. The variant with the highest mAP is highlighted in bold. Detected features: OD; MA; and HEMO.

Models	AP			mAP
	OD	MA	HEMO	
nano	0.965	0.224	0.544	0.578
**small**	**0.982**	**0.265**	**0.506**	**0.584**
medium	0.971	0.212	0.489	0.557
large	0.966	0.192	0.527	0.562
extra-large	0.973	0.247	0.479	0.566

**Table 5:** Results obtained by each variants of YOLOv8’s models, with the metrics AP and mAP for the IoU threshold of 0.5 in the validation dataset excluding the microaneurysms class. The variant with the highest mAP is highlighted in bold. Detected features: OD; and HEMO.

Models	AP		mAP
	OD	HEMO	
nano	0.974	0.491	0.733
**small**	**0.982**	**0.555**	**0.769**
medium	0.983	0.491	0.737
large	0.969	0.469	0.719
extra-large	0.979	0.470	0.724

**Table 6:** Results obtained by each variants of YOLOv8’s models, with the metrics AP and mAP for the IoU threshold of 0.5 in the validation dataset. The variant with the highest mAP is highlighted in bold. Detected features: MA; HEMO; and EX.

Models	AP			mAP
	MA	HEMO	EX	
**nano**	**0.182**	**0.418**	**0.417**	**0.339**
small	0.173	0.420	0.352	0.315
medium	0.189	0.483	0.344	0.339
large	0.206	0.474	0.319	0.333
extra-large	NA	NA	NA	NA

**Table 7:** Results obtained by model *c* and *e* from YOLOv9, with the metrics AP and mAP for the IoU threshold of 0.5 in the validation dataset. The variant with the highest mAP is highlighted in bold.

Models	AP			mAP
	MA	HEMO	EX	
compact	0.212	0.484	0.378	0.358
**extended**	**0.205**	**0.484**	**0.390**	**0.359**

**Table 8:** Results obtained with the metrics: mAP, AP, Precision, Recall, Accuracy, and F1 score with the best models from our experiment with YOLOv8 and v9 during the validation step.

Models	AP				mAP	P	Re	Acc	F1
	OD	MA	HEMO	EX					
Test YOLOv8-small	0.982	0.265	0.506	NA	0.584	0.758	0.405	0.359	0.528
Test YOLOv8-small	0.982	NA	0.555	NA	0.769	0.716	0.650	0.517	0.682
Test YOLOv8-nano	NA	0.182	0.418	0.417	0.339	0.590	0.216	0.188	0.316
Test YOLOv9-extended	NA	0.205	0.484	0.390	0.359	0.610	0.239	0.208	0.344

**Table 9:** Results obtained with the metrics mAP and AP during the validation stage with the best models from our experiment (highlighted in bold) involving YOLOv8 and v9 compared with related works from the literature.

Models	Datasets	AP				mAP
		OD	MA	HEMO	EX	
YOLOv3 [30]	Custom—1959 Retina Images in the training step; 204 retina images in the test step	0.990	NA	NA	NA	0.990
ResNet [30]		0.956	NA	NA	NA	0.956
DenseNet [30]		1.000	NA	NA	NA	1.000
YOLOv5 [50]	STARE, AIRA, G1020, and MESSIDOR2—5823 training images—6372 testing images from seventeen datasets	0.996	NA	NA	NA	0.996
YOLOv7 [51]	DRISHTI-GS and ORIGA—68 training images and 33 testing images	0.995	NA	NA	NA	0.995
HED [31]	DDR—757 fundus images with DR lesion annotations	NA	0.060	0.189	0.271	0.170
DeepLab-v3+ [31]		NA	0.039	0.405	0.438	0.332
SSD [31]		NA	0.000	0.000	0.000	0.006
YOLO [31]		NA	0.000	0.010	0.004	0.004
Faster RCNN [31]		NA	0.009	0.087	0.030	0.093
YOLOv4+SGD [52]		NA	0.019	0.085	0.037	0.072
YOLOv5 [32]		NA	0.005	0.130	0.031	0.104
YOLOv8 SGD+Tilling [32]		NA	0.105	0.333	0.229	0.249
YOLOv8 Adam opt+Tilling [32]		NA	0.111	0.352	0.224	0.2630
YOLOv3+Adam opt+dropout [49]		NA	NA	NA	NA	0.216
Mask R-CNN Adam opt [53]		NA	0.109	0.258	0.260	0.235
Mask R-CNN Adam opt + Tilling [53]		NA	0.164	0.352	0.294	0.290
YOLOv2 [48]	EyePacs—5000 annotated images for training—519 image as test	NA	0.813	NA	NA	0.813
YOLOv3 [54]	Private dataset from Amar Eye Hospitl and Kaggle-DR dataset	NA	0.844	0.920	NA	0.922
YOLOv2 [54]		NA	0.814	0.905	NA	0.906
YOLOv1 [54]		NA	0.789	0.899	NA	0.894
**test YOLOv8-s**	**100 manually annotated images from Messidor—4256 images after augmentation**	**0.982**	**0.265**	**0.506**	**NA**	**0.584**
**test YOLOv8-s**		**0.982**	**NA**	**0.555**	**NA**	**0.769**
**test YOLOv8-n**		**NA**	**0.182**	**0.418**	**0.417**	**0.339**
**test YOLOv9-e**		**NA**	**0.205**	**0.484**	**0.390**	**0.359**

Definitions: NA, Not Available; YOLO, You Only Look Once, version from 1 to 9; ResNet, Residual neural network; DenseNet, Densely connected neural network; HED, Holistically-nested Edge Detection; SSD, Single Shot Multi-Box Detector; Mask R-CNN, Mask Regions with Convolutional Neural Network; SGD, Stochastic Gradient Descent; Adam opt, Adam optimizer.

## Data Availability

We used publicly available dataset in this study. Messidor dataset at doi:10.5566/ias.1155. The annotated image dataset created for this research is available on request.
